# Does Meconium Peritonitis Pseudo-Cyst Obstruct Labour?

**DOI:** 10.1155/2012/593143

**Published:** 2012-06-07

**Authors:** Khalil Al Tawil, Walid Salhi, Safiah Sultan, Mohammad Namshan, Saeed Mohammed

**Affiliations:** ^1^Department of Paediatrics, King Abdulaziz Medical City, P.O. Box 22490, Riyadh 11426, Saudi Arabia; ^2^Department of Gynaecology and Obstetrics, King Abdulaziz Medical City, P.O. Box 22490, Riyadh 11426, Saudi Arabia; ^3^Department of Surgery, King Abdulaziz Medical City, P.O. Box 22490, Riyadh 11426, Saudi Arabia; ^4^Department of Radiology, King Abdulaziz Medical City, P.O. Box 22490, Riyadh 11426, Saudi Arabia

## Abstract

Meconium peritonitis pseudo-cyst is very rare. Its perinatal management is controversial and can be associated with increased fetomaternal morbidity and mortality. A 34-week gestation infant with large meconium peritonitis pseudo-cyst, detected by intrapartum fetal ultrasound study, had abnormally increased ratio of fetal abdominal circumference to head circumference. Intrapartum aspiration of the pseudo-cyst was performed and was followed by a smooth vaginal delivery. The postnatal course of the baby showed that early laparotomy was essential for stabilisation of the infant's general condition.

## 1. Introduction

Obstruction of labour due to fetal causes is quite rare. Fetal macrosomia accounts for majority of cases, while few other causes were reported [1, 2].

Meconium peritonitis (MP) is an aseptic peritonitis caused by spread of meconium into the fetal peritoneal cavity after an intestinal perforation [3, 4]. The prevalence of MP is about 0.29 per 10,000 live births; mortality ranges from 2.4% to 50%, and morbidity up to 34% [5, 6].

Meconium peritonitis pseudo-cyst (MPC) accounts for 12% of MP [5], it is an extra luminal collection of meconium surrounded by fibrotic membranes [7]. MPC is not counted in the causes of obstruction of labour.

We are reporting the successful antenatal and postnatal management of a male baby born at 34 weeks of gestation with large MPC secondary to jejunal perforation.

## 2. Case Presentation

**Figure 1:**
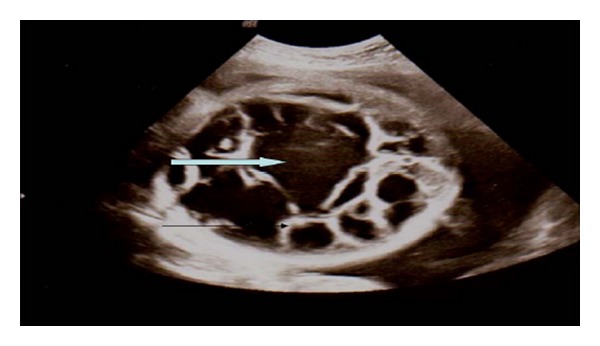
Antenatal ultrasound scan view showing the foetal abdomen and revealed the presence of a large cystic lesion with multiple thick septation (black arrow) containing fluid and fine echoes (block arrow).

A 24-year-old primigravid mother presented at 34 weeks of gestation with labour contractions, polyhydramnios, and prolonged rupture of membranes for 3 days. The pregnancy was unbooked at our hospital. She was commenced on systemic antibiotics. Intrapartum ultrasound (US) revealed a male fetus, cephalic presentation, had large intra-abdominal cystic mass with echogenic septation, full of hypoechoic debris (Figure 1). The mass measured 13 × 12 cm in its cross-section, fetal abdominal circumference (AC) was 421 mm (normal at 34 wks is 304 mm ±10 mm), and head circumference (HC) was 320 mm (normal at 34 wks is 305 ± 25 mm).

**Figure 2:**
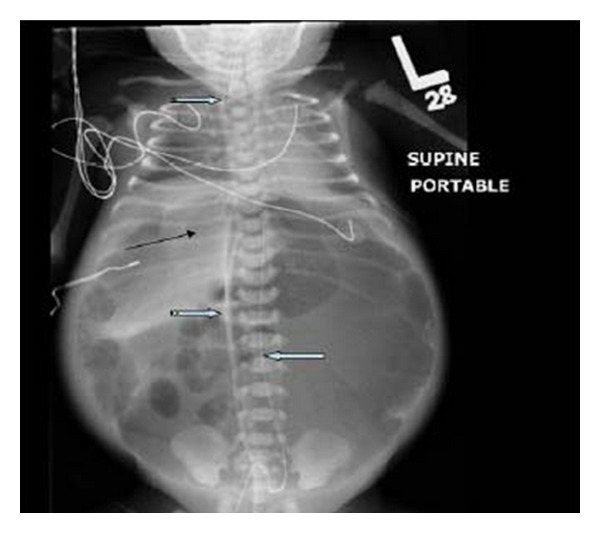
A baby gram X-ray showing a severely distended abdomen with free intraperitoneal air in front of liver (black arrow) and umbilical artery catheter (right striped block arrow) and umbilical vein catheter (left block arrow) in situ. An endotracheal tube (notched block arrow) is noted in situ and both diaphragms are significantly elevated.

**Figure 3:**
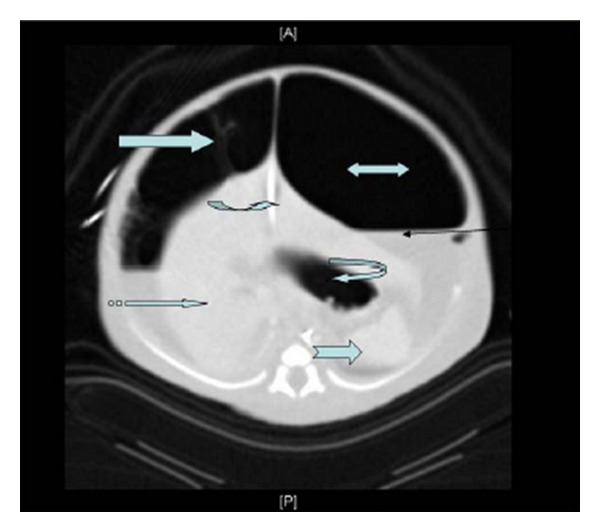
Abdominal CT scan image: showing abundant intraperitoneal space (biheaded block arrow) and fluid levels (black line arrow) with multiple thick septation (right block arrow) noted mainly at the right hypochondrium. Umbilical vein catheter (curved right arrow), spleen (notched right block arrow), stomach (curved left arrow), and liver (striped right block arrow) were noted.

Fetal Doppler study of the umbilical arteries was abnormal; therefore, labour was allowed to proceed. In view of the fetal AC being much larger than HC and the possibility of obstruction of labour by the large abdomen, transabdominal aspiration of the MPC was performed using a long, spinal needle gauge 20; 420 mL of meconium were aspirated. Labour progressed well and smooth vaginal delivery of a live male baby occurred 4 hours later. He weighed 2330 grams with good Apgar score. Shortly after birth, he developed respiratory distress and abdominal distension, so he was transferred to the neonatal intensive care unit, where he was supported by a mechanical ventilator, intravenous fluids and inotropes. US-guided needle aspiration of the MPC was performed, obtaining 120 mL of greenish stained fluid. The baby's general condition continued to be critical, and the abdominal distension did not subside but rather worsened. A plain abdominal radiograph (Figure 2) showed significant pneumoperitoneum. Abdominal CT (Figure 3) was performed at age of 48 hours and showed free air and large, loculated collection of thick debris at the left side of upper abdomen.

Emergency laparotomy revealed a pseudo-cyst filled with air and meconium occupying the entire anterior and upper abdomen, isolated jejunal volvulus with a normal bowel orientation and a long-existing ischemic jejunal perforation. An 18 cm segment of jejunum was resected, and primary anastomosis was performed. Two days post-surgery, the baby was successfully weaned from mechanical ventilation and inotropes.

The results of DNA testing for cystic fibrosis (CF) mutations and culture of drained abdominal fluids were negative. Enteral feeding was initiated after the bowel been rested for 10 days. Initially; he had feeding intolerance that eventually subsided. The baby was discharged home in good health at the age of 7 weeks. Postdischarge followup at age of 10 months showed a healthy growing infant.

## 3. Discussion

The availability of high-resolution imaging equipments facilitated the perinatal diagnosis and management of most cases of MP and MPC [4, 5, 7]. The differential diagnosis of CMP includes haemangioma, teratoma, ovarian dermoid, hepatoblastoma, and metastatic neuroblastoma [8].

The underlying cause of meconium pseudocyst is an ischemic bowel perforation leaking meconium. It can be associated with bowel atresia, midgut volvulus, or both conditions [5].

Once an antenatal diagnosis of complicated MP or MPC is reached, the mother should be referred to a tertiary care centre where close monitoring is possible and facilities for urgent perinatal intervention are available if needed [4, 9].

At approximately 34 weeks of gestation, HC and AC are expected to be equal or near equal. In our case, the finding on intrapartum US imaging of fetal AC to be much larger than HC was unusual. If fetal paracentesis was not performed, the head would have been delivered vaginally but the abdomen might got stuck inside, endangering the life of the fetus and his mother. Such risks must always be considered [9, 10].

Previous reports have proposed cesarean delivery (CS) of fetuses with MPC [4, 5, 9]. CS of a fetus with a severely distended abdomen is difficult and risky. Such risk would be minimized by fetal paracentesis and CS would not be necessary in most cases [11].

Few cases of MPC were reported to have intrapartum fetal paracentesis prior to vaginal delivery [6, 11], but previous reports lack the guidance of fetal measurements of HC and its relation to AC.

The association between fetal MP and midgut volvulus is highly suggestive of CF and parents should be counselled regarding that possibility [5]. CF is an unusual disease in the Arabian community [12].

Similar to our infant, the postnatal clinical course of MPC is usually characterised by cardiorespiratory failure due to pulmonary hypertension, prolonged abdominal distension [6, 7]. Laparotomy is necessary to achieve infant's stability. It controlled the increased intra-abdominal pressure and improved respiratory and cardiovascular functions as reported by others [7, 13]. Therefore, in concordance with other's suggestions, early postnatal surgery of the infant is strongly advisable and can be associated with low mortality [13–15].

## 4. Conclusion

Large MPC associated with fetal AC greater than HC may obstruct labour, and intrapartum fetal paracentesis has to be seriously considered to ensure smooth delivery. Fetal MPC does not determine the mode of delivery. Immediate postnatal paracentesis and early laparotomy are associated with rapid neonatal recovery.

##  Conflict of Interest

The authors declare no competing financial interests in relation to their paper.
